# Cumulative pre-cooling methods do not enhance cycling performance in tropical climate

**DOI:** 10.1371/journal.pone.0291951

**Published:** 2023-10-12

**Authors:** Aurélie Collado, Kévin Rinaldi, Eric Hermand, Olivier Hue

**Affiliations:** 1 Université des Antilles, ACTES (UPRES EA 3596), UFR STAPS, Pointe-à-Pitre, France; 2 Arkea Samsic Pro Cycling Team, Bruz, France; 3 Univ. Littoral Côte d’Opale, Univ. Artois, Univ. Lille, CHU LIlle, ULR 7369—URePSSS—Unité de Recherche Pluridisciplinaire Sport Santé Société, Dunkerque, France; United States Olympic & Paralympic Committee, UNITED STATES

## Abstract

The main objective of this study was to investigate the effect of mixed cooling techniques (combination of internal and external strategies, with and without menthol) during warm-up for a time trial in tropical climate. Seven heat-acclimatized trained male road cyclists participated in three experimental sessions consisting of 20-min cycling performances on a velodrome track in ecological hot and humid conditions (Guadeloupe, French West Indies; WBGT: 27.64±0.27°C; relative humidity: 76.43±2.19%), preceded by a standardized 30-min warm-up and the ingestion of cold menthol water (1) with a cooling vest soaked in ice water (ICE-VEST), (2) with a cooling vest soaked in ice menthol water (MEN-VEST), and (3) without a vest (NO-VEST). Cycling performance (total distance, distance traveled per 2-min block), physiological parameters (core body temperature recorded, heart rate) and perceptions (exertion, thermal comfort, thermal sensation) were assessed. No between-condition differences were found for physiological parameters, the total covered distance or the distance traveled per 2-min block. However, distance traveled per 2-min decreased with time *(p = 0*.*03)*, with no difference between conditions, suggesting a variation in pace during the cycling performance trial (e.g., mean±SD: 1321±48.01m at T2; 1308±46.20m at T8, 1284±78.38m at T14, 1309±76.29m at T20). No between-condition differences were found for perception of exertion, thermal comfort and thermal sensation during the warm-up (11.83±3.34; 2.58±1.02; 4.39±0.94, respectively) and the performance (17.85±0.99; 2.70±1.25; 5.20±1.20, respectively) but the pairwise comparisons within condition revealed a significant increase of TS values from T0 (4.57±1.13) to T20 (6.00±0.58) only in NO-VEST condition (*p =* 0.04). The absence of modification of thermal sensation at the end of the cycling test under the mixed conditions (ICE-VEST and MEN-VEST) suggests a beneficial effect of wearing a cooling vest on thermal sensation although it had no effect on performance.

## Introduction

The performance of intense exercise in a tropical climate (i.e., hot and wet climate) increases the exercise-induced stress on the cardiovascular and thermoregulatory systems, and severely impacts aerobic performance [1–4]. The literature has shown the relevance of cooling strategies prior to exercise (pre-cooling) to limit the negative impact on aerobic performance [5–8]. Among these countermeasures, external (e.g., cooling vest, water immersion) and internal (e.g., drinking cold water/ice-slurry solution) cooling strategies mainly restrict the rise in body temperature and thereby increase the time to exhaustion [5, 8–11]. Wearing a cooling vest before exercise helps limit the core temperature increase [7, 8, 12, 13] and improves the rating of perceived exertion (RPE) and thermal perceptions [13, 14]–which, as behavioral controllers [15] can lead to a decrease in performance through an anticipatory and voluntary down-regulation of effort [16], thereby improving performance [6, 8, 12]. Pre-cooling ingestion strategies have shown beneficial effects on endurance capacity and performance [9, 17, 18], dependent on the temperature of the ingested solution [9, 18–21]. In addition, recent studies were focused on menthol, a compound of plant origin (Mentha), which acts on the TRPM8, TRPA1, and TRPV3 thermoreceptors [22], hence modifying inner and cutaneous thermal perceptions. A low dose of menthol used would activate the TRPM8 receptor which is usually activated by temperatures below 25°C [22]. For example, a cooling sensation similar to the action of spraying cold-water on the face is provoked when menthol is applied to skin and mucosal surfaces [23]. In sport performance, several studies have clearly revealed the potentiating effect of menthol on cold solutions when they are ingested [18, 19, 24, 25] or applied [18, 26, 27]. Among these strategies, the ingestion of cold water or ice-slush with a menthol aroma was proven to be the most effective beverage for endurance exercise in a tropical climate: the colder the drink, the greater the effects [19].

According to a recent review on the ergogenic effects of pre-cooling in highly trained athletes [28], the best option for enhancing the benefits of pre-cooling strategies is to mix external and internal cooling. Indeed, it has been shown that the cumulative effect of two pre-cooling strategies enhances the cooling efficacy [7, 29, 30], and a combination of pre-cooling and water ingestion led to an increase in exercise endurance in a hot and humid environment [31]: the combination of cooling strategies enhanced heat storage capacity and decreased the cardiovascular and thermoregulatory strains. Nevertheless, it should be noted that the pre-cooling method used in this study consisted in an immersion in a 25°C water for 30 min. Although immersion, leading to a large and rapid reduction of core body temperature [32], seems to be the most effective pre-cooling strategy [5, 10, 18], it is time-consuming and logistically very difficult to use in real sports contexts [2, 33]. To overcome this issue, cooling vests, when sufficiently in contact with the skin (i.e., with numerous thermoreceptors) gave similar results [32]. Recently, a study concluded that the ice vests seem to be the most adopted pre-cooling strategy by elite athletes but also the only one minimizing core temperature during the warm-up [34]: pre-cooling with a cooling vest for 20 min at rest before exercising increased endurance performance more than a 20-min warm-up [12], but athletes need to warm up, and scheduling a time for immersion beforehand lengthens their preparation and is not always practicable. Some experimentations tried to circumvent this problem by associating the cooling strategies with the warm-up, showing that warming up with a cooling vest limits the increase in core temperature [13, 14] and the thermal discomfort [13, 14], and improves aerobic performance [14].

Given the potentiating effect of menthol [18] and the ergogenic effect of mixed pre-cooling strategies [28] on aerobic performance, the main objective of this study was to investigate the added effect of mixed techniques (a combination of internal and external strategies, with and without menthol) during the warm-up on the aerobic performance (i.e., a 20-min cycling performance described as a time trial) in a tropical climate. The aim was to determine, in the tropical climate, (1) whether combined internal pre-cooling with menthol and external pre-cooling with or without menthol during a warm-up would improve cycling performance, perceptions (i.e., thermal environment, RPE) and core temperature of athletes more than only internal pre-cooling with menthol; and (2) whether, in the mixed conditions, adding menthol to the external pre-cooling would improve the cycling performance, perceptions and core temperature.

## Materials and methods

### Participants

Seven heat-acclimatized (i.e., living and training in Guadeloupe) trained male road cyclists (age: 29±8 years, weight: 67±8 kg, maximal aerobic power: 375±65 watts) were recruited among Guadeloupean cycling teams. They were well-trained and were regularly competing in elite road races. These cyclists were training at least 15 hours per week at the time of the study (weekly training 17±2 hr). All completed a medical screening questionnaire and gave written informed consent prior to the study, which was approved by the Ethics Committee of the Training and Research in Sport Science Unit in Guadeloupe (Ministry of Higher Education and Research) and conducted according to the Declaration of Helsinki.

The cyclists were instructed to avoid training sessions for 24 hr prior to testing and were allowed 60 min of light-intensity exercise 48 hr before each experimental session. Caffeine, drugs and alcohol were strictly prohibited for 24 hr before the start of the experimental session. The cyclists were instructed to consume the same meals each day before each session. Whatever the time of passage, the cyclists were asked to have, 2 hours before, the same standardized French breakfast (bread, butter, jam, yogurt) and 500 mL of water at ambient temperature. For each participant, the sessions began at the same time of day to remove any effect of circadian variation (between 10:00 a.m. and 2:00 p.m.).

### Procedure

Participants performed trials under three randomized experimental conditions of pre-cooling—separated by one week—followed each time by a cycling performance trial on an outdoor velodrome in ecological hot and humid conditions (Guadeloupe, French West Indies; WBGT: 27.7±0.3°C; relative humidity: 76±2%). The cyclists completed each entire experimental session on their personal road cycling bike, using standardized equipment (no extension, classic helmet, racing clothing during the cycling performance). The three experimental sessions consisted of a standardized warm-up associated with one of the following randomized cooling conditions: (1) ingestion of cold menthol water + wearing a cooling vest soaked in ice water (ICE-VEST), (2) ingestion of cold menthol water + wearing a cooling vest soaked in ice menthol water (MEN-VEST), and (3) ingestion of cold menthol water without wearing a vest (NO-VEST). The cooling vest covered the entire torso and back. Each experimental session included (1) a 30-min warm-up on a cycle trainer (Tacx Satori T1856 Tacx BV, Wassenaarn, The Netherlands) with pre-cooling strategies, and (2) the cycling performance on the track. The warm-up consisted of a gradual increase in intensity in such a way as to resemble as much as possible the protocols used by the athletes when competing in time trial events: 10-min at i2, 10-min at i3, 3-min at i4, 2-min at i5 and 5-min at i2 ([35] for intensity chart). A standardized transition of 5 min between the end of the warm-up and the beginning of the cycling performance test gave the athletes time to dress in their complete outfits, take their bikes out of the home-trainer, and get to the starting line. During the cycling performance, they had no information that could help them manage the pace and were only informed when they had reached the last 5 minutes and the last minute of the 20-minute test.

During pre-cooling, taking place during the warm-up, the cyclists ingested a total of 8 g.kg^-1^ of cold menthol water (3°C) divided into three intakes every 15 min at T0, T15 and T30. The menthol (L-menthol) beverage was a 0.025% natural menthol aroma (% vol: 86.0±1.0%; dosage: 0.5 g.L^-1^) (Robertet, Grasse, France; see [20] for procedure) whose concentration has previously been shown to be beneficial in tropical climate during efforts between 20 min and 1h30 [19, 20]. The cooling vest was kept cool for the duration of the warm-up by soaking it every 10 min (T0, T10 and T20) in ice water (3°C) for the ICE-VEST condition or ice water + menthol for the MEN-VEST condition (4°C; dosage: 1%); the vest was adjusted to the body with belts to optimize contact surface with the body. The temperature of each beverage and the ice water for the soaked vest was measured with a digital thermometer (YSI 409B, Yellow Springs Instruments, OH, USA).

After the warm-up with the pre-cooling strategies, the cyclists covered the greatest distance possible in 20 min on an open-air velodrome track.

### Assessment

The cycling performance on the velodrome track was measured as the total distance traveled (m) over 20 min, and the distance traveled (m) per 2-min block was also recorded. The velodrome measured 333m and was marked every 5m. We measured the final distance traveled between marks with a tape meter, thus enabling a precise performance report (i.e., near 50 cm).

Heart rate (HR) was continuously monitored during the entire experimental session using a portable telemetry unit (Suunto Memory Belt, Suunto, Vantaa, Finland), and averaged by 1-min blocks for the warm-up and 2-min blocks for the cycling performance. Core temperature (i.e., gastrointestinal temperature-Tgi) was recorded every 5 min during warm-up and at the beginning and end of the cycling performance using ingestible temperature measurement pills (CorTemp, HQ, Inc., Palmetto, FL, USA). To ensure the pill was out of the stomach and thereby avoid Tgi variability due to pill movement or fluid/food consumption, the athletes were invited to ingest these pills 6 hr before all experimental sessions. The cooling rate which corresponds to a temperature difference (∆Tgi) was calculated as the difference in Tgi from pre- to post-warm-up and from pre- to post-cycling performance.

Perception was measured every 5 min during warm-up: from T0 to T30 for thermal comfort (TC) and thermal sensation (TS), and from T5 to T30 for RPE. For the cycling performance, TC and TS were recorded at the beginning and the end of the cycling performance and RPE at the end. The perception of exertion was evaluated with the 15-point Borg’s RPE scale [36] that ranges from 6 (no exertion) to 20 (maximal exertion). Regarding the perception of the thermal environment, participants were asked to rate their perceived TC and TS on two continuous rating scales ranging from, respectively, 1 (“not uncomfortable”) to 4 (“very uncomfortable”), and from 1 (“slightly cool”) to 7 (“extremely hot”) [37].

### Statistical analyses

Kolmogorov–Smirnov and Levene tests were used to check the normality of distribution and the homogeneity of variance, respectively. When the assumption of normality of distribution and/or homogeneity of variance was violated, non-parametric analyses were conducted to determine whether differences existed between periods and conditions. Accordingly, a Friedman ANOVA and a Wilcoxon test were used for the TS, the TC and the RPE scores collected during the warm-up to assess whether differences existed between T0, T5, T10, T15, T20, T25 and T30 (T5, T10, T15, T20, T25 and T30 for RPE scores) for all conditions (i.e., ICE-VEST, MEN-VEST, NO-VEST) and between the conditions for all 5-min blocks. Similar statistical analyses were conducted for these scales during the cycling performance to assess (1) condition differences before the cycling performance for TS and TC and after the cycling performance for TS, TC and RPE, and (2) differences of TS, TC and RPE scores before and after the cycling performance within each condition.

For the other variables, repeated measures ANOVAs were globally conducted with Condition as the between-factor and Period as the within-factor. For all statistical analyses below, post-hoc analyses were conducted with Tukey’s HSD test when the application conditions were respected.

HR averaged for each 2-min block was analyzed with repeated measures ANOVAs with Condition (i.e., ICE-VEST, MEN-VEST, NO-VEST) as the between-factor and Period (i.e., every 2 minutes from T0 to T30 for the warm-up and from T0 to T20 for the cycling performance) as the within-factor.

Similar statistical analyses were conducted for the distance traveled (m) per 2-min block with Condition (i.e., ICE-VEST, MEN-VEST, NO-VEST) as the between-factor and Period (i.e., every 2 min from T0 to T20) as the within-factor.

The total distance traveled (m) over 20 min during the cycling performance and the ∆Tgi (during the warm-up and cycling performance) were analyzed with repeated measures ANOVAs with Condition (i.e., ICE-VEST, MEN-VEST, NO-VEST).

Data analyses were conducted using Statistica 12.0 (StatSoft). For all statistical analyses, alpha was set at .05 and effect sizes (*η*_*p*_^2^) are indicated.

## Results

### Cycling performance

**Fig 1:**
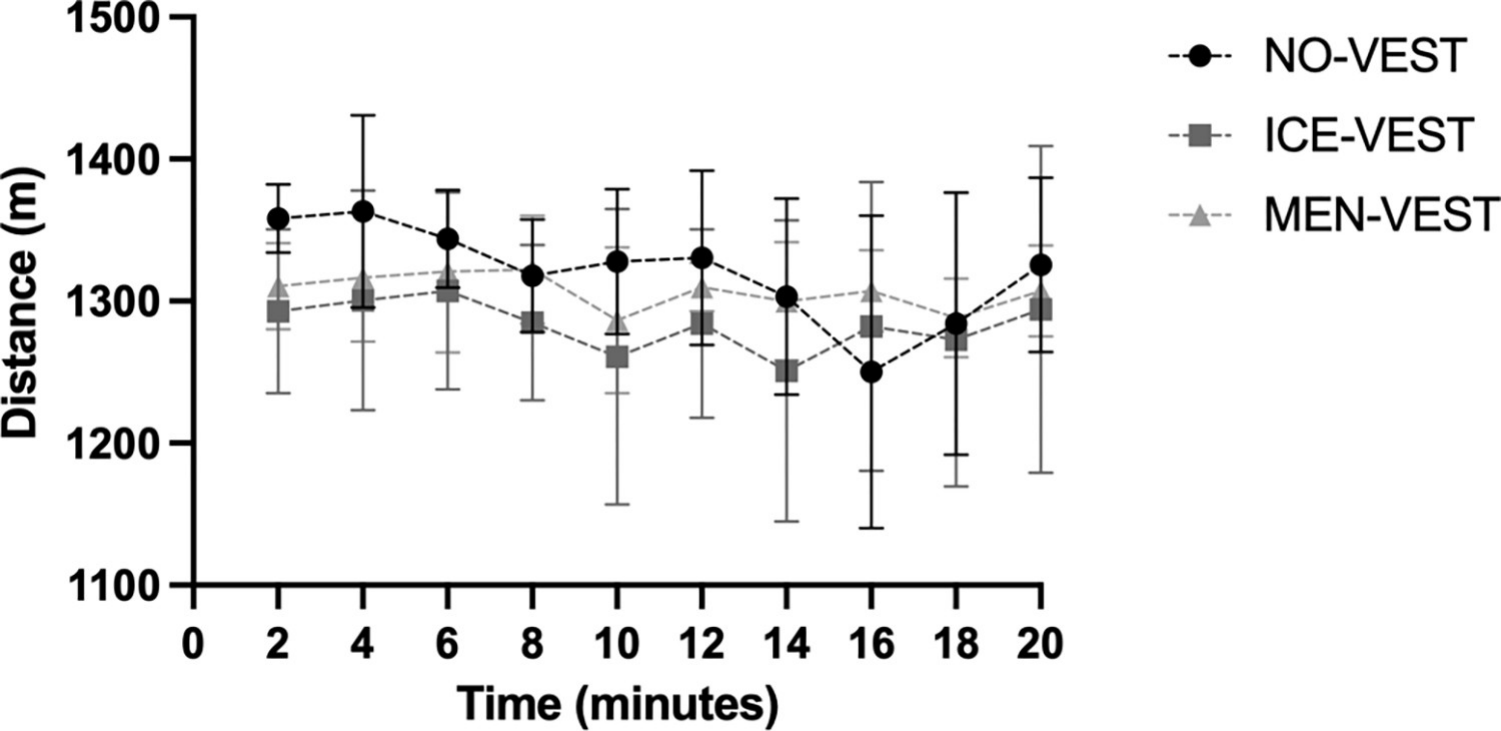
Distance traveled by block of 2-min according the conditions. NO-VEST: without vest, ICE-VEST: cooling water vest and MEN-VEST: cooling menthol vest. The error bars represent standard deviation from the mean.

For the total covered distance, there was no significant effect of Condition [*F*(2,10) = 0.41, *p* = 0.68, *η*_*p*_^2^ = 0.08] (Table 1). Regarding the distance traveled per 2-min block (Fig 1), although no significant effect was found for either Condition [*F(*2,10) = 0.41, *p* = 0.68, *η*_*p*_^2^ = 0.08] nor the Condition×Period interaction [F(18,90) = 1.57, *p* = 0.09, *η*_*p*_^2^ = 0.24], statistical analyses showed a significant main effect of Period [*F*(9,45) = 2.34, *p* = 0.03, *η*_*p*_^2^ = 0.32].

**Table 1:** Means, standard deviations (SD) and confidence intervals (CI) for the total distance traveled.

	NO-VEST	ICE-VEST	MEN-VEST
**Total distance (m)** **(mean±SD**)	13204±500	12830±771	13068±264
95% CI	☯12742; 13666]	☯12117; 13543]	☯12791; 13345]

NO-VEST: without vest, ICE-VEST: cooling water vest and MEN-VEST: cooling menthol vest.

### Cooling rate

**Fig 2:**
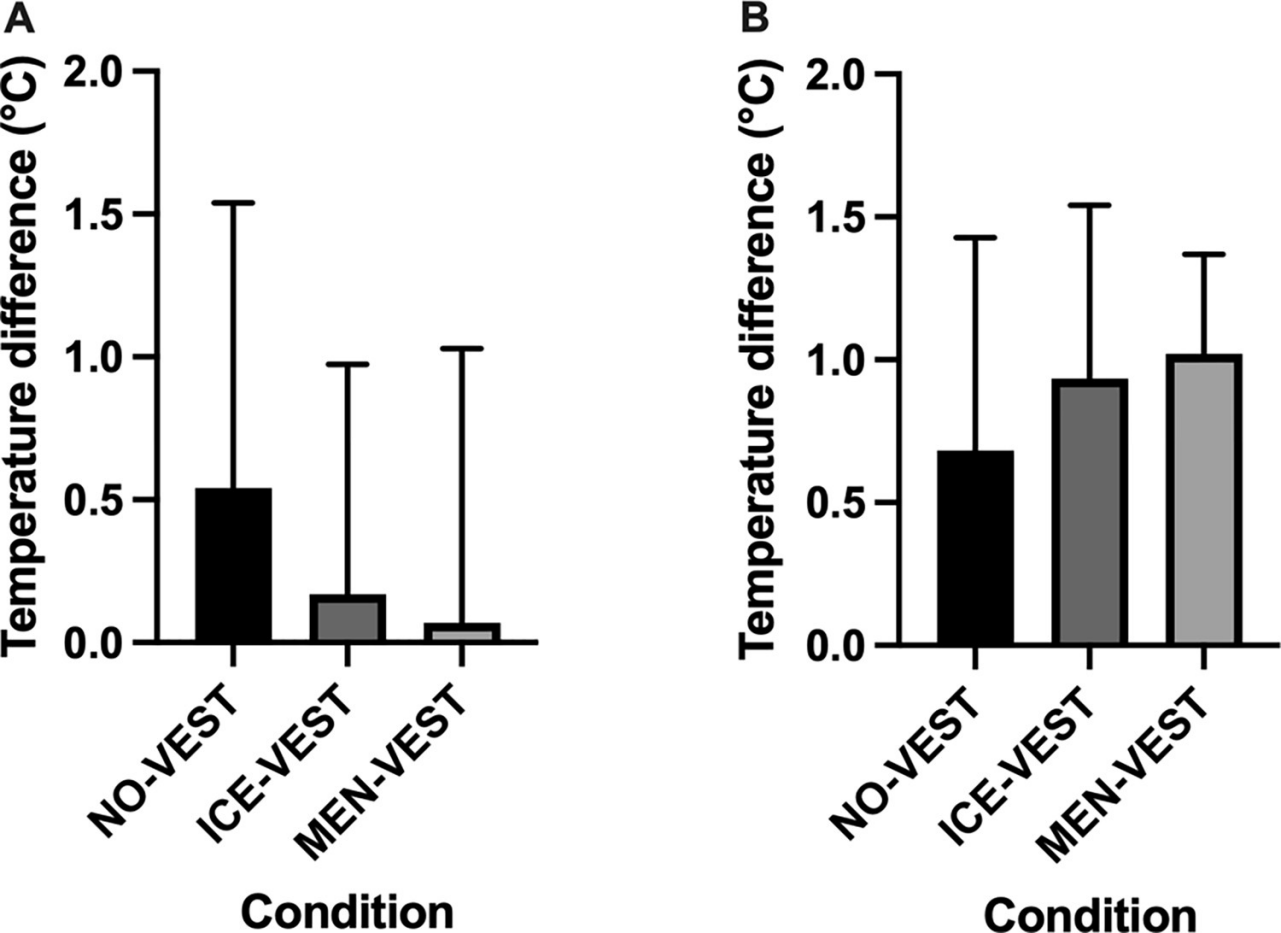
Comparison of the cooling rate between conditions. NO-VEST: without vest, ICE-VEST: cooling water vest and MEN-VEST (cooling menthol vest) during the warm-up (A) and the cycling performance (B). The error bars represent standard deviation from the mean.

The statistical analyses of the cooling rate with ∆Tgi showed no inter-condition differences during the warm-up [*F*(2,10) = 0.81, *p* = 0.47, *η*_*p*_^2^ = 0.14] or cycling performance [*F*(2,6) = 1.24, *p* = 0.36, *η*_*p*_^2^ = 0.29] (Fig 2).

### Heart rate

**Fig 3:**
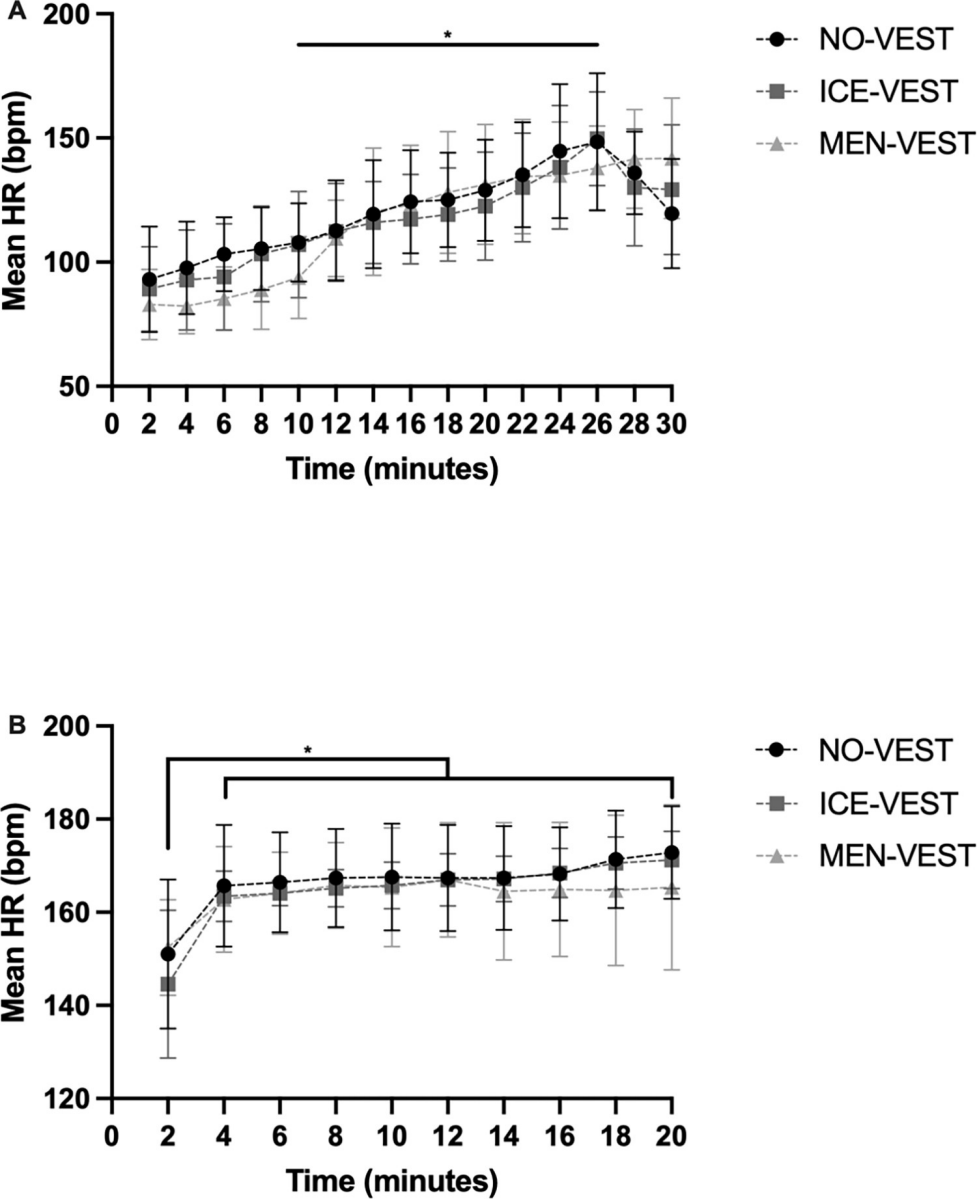
Comparison of the mean heart rate between conditions. NO-VEST: without vest, ICE-VEST: cooling water vest and MEN-VEST: cooling menthol vest during the warm-up (A) and the cycling performance (B). The error bars represent standard deviation from the mean. Significant differences for time effects have been marked as follows: *p<0.05.

For HR (Fig 3), whereas no significant effect of Condition [*F*(2,8) = 0.05, *p* = 0.096, *η*_*p*_^2^ = 0.01] was observed during the warm-up, repeated measures ANOVA revealed a significant main effect of Period [*F*(14,56) = 48.95, *p*<0.0001, *η*_*p*_^2^ = 0.93] and the Condition×Period interaction [*F*(28,112) = 1.76, *p* = 0.02, *η*_*p*_^2^ = 0.31]. Tukey’s post hoc analysis, on the main effect of Period and Condition×Period interaction, indicated a continuous increase in mean HR during the warm-up for all conditions, from T10 to T26, followed by a decrease to T30 in both conditions (Fig 3A). For the cycling performance (Fig 3B), a significant main effect of Period [*F*(9,45) = 21.94, *p*<0.0001, *η*_*p*_^2^ = 0.81] was observed. There was no effect of either Condition [*F*(2,10) = 1.00, *p* = 0.40, *η*_*p*_^2^ = 0.17] or the Condition×Period interaction [*F*(18,90) = 1.14, *p* = 0.33, *η*_*p*_^2^ = 0.19].

### Rating of perceived exertion

During the warm-up with pre-cooling strategies (Table 2), a Friedman ANOVA indicated significant differences between RPE’ measures from T5 to T30 for ICE-VEST (Friedman χ2 = 31.18; df = 5; *p*<0.001), MEN-VEST (Friedman χ2 = 26.28; df = 5; *p*<0.001) and NO-VEST (Friedman χ2 = 32.33; df = 5; *p*<0.001). The pairwise comparisons of RPE scores revealed a continuous increase from T5 to T25 followed by a decrease from T25 to T30 in all conditions (data not represented graphically). No inter-condition differences were found for T5 (Friedman χ2 = 1.63; df = 2; *p* = 0.44), T10 (Friedman χ2 = 1.50; df = 2; *p* = 0.47), T15 (Friedman χ2 = 3.52; df = 2; *p* = 0.17), T20 (Friedman χ2 = 0.64; df = 2; *p* = 0.73), T25 (Friedman χ2 = 0.90; df = 2; *p* = 0.64) or T30 (Friedman χ2 = 1.20; df = 2; *p* = 0.55).

**Table 2:** Medians and interquartile ranges (IQR) for the rating of perceived exertion during the cycling performance.

	NO-VEST	ICE-VEST	MEN-VEST
**Rating of Perceived Exertion** Median (IQR)	18.00 (17.00–18.00)	18.00 (17.00–19.00)	18.50 (17.25–19.00)

NO-VEST: without vest, ICE-VEST: cooling water vest and MEN-VEST: cooling menthol vest.

For the cycling performance (Table 3), a Friedman ANOVA indicated no between-condition differences of RPE at the end of the performance (Friedman χ2 = 1.68; df = 2; *p* = 0.43) (Table 2).

**Table 3:** Medians and interquartile ranges (IQR) for the thermal sensation (TS) and the thermal comfort (TC) during the warm-up and the cycling performance according the conditions.

	NO-VEST	ICE-VEST	MEN-VEST
**TS (warm-up)** (Median, IQR)	4.00 (4.00–5.00)	4.00 (4.00–5.00)	4.00 (4.00–5.00)
**TS (performance)** (Median, IQR)	6.00 (4.00–6.00)	4.50 (4.00–6.00)	6.00 (4.00–6.00)
**TC (warm-up)** (Median, IQR)	3.00 (2.00–4.00)	3.00 (2.00–3.00)	2.00 (2.00–3.00)
**TC (performance)** (Median, IQR)	4.00 (1.75–4.00)	2.50 (1.00–3.25)	3.00 (1.25–4.00)

NO-VEST: without vest, ICE-VEST: cooling water vest and MEN-VEST: cooling menthol vest.

### Thermal sensation and thermal comfort

A Friedman ANOVA indicated significant differences between TS values from T0 to T30 for ICE-VEST (Friedman χ2 = 23.88; df = 6; *p*<0.001), MEN-VEST (Friedman χ2 = 14.73; df = 6; *p* = 0.02) and NO-VEST (Friedman χ2 = 17.45; df = 6; *p*<0.01) during the warm-up (Table 3). The pairwise comparisons of TS scores revealed a continuous increase from T0 to T25 followed by a decrease from T25 to T30 in all conditions (data not represented graphically). No inter-condition differences were found for T0 (Friedman χ2 = 4.77; df = 2; *p* = 0.09), T5 (Friedman χ2 = 1.40; df = 2; *p* = 0.50), T10 (Friedman χ2 = 0.88; df = 2; *p* = 0.65), T15 (Friedman χ2 = 3.50; df = 2; *p* = 0.17), T20 (Friedman χ2 = 2.00; df = 2; *p* = 0.37), T25 (Friedman χ2 = 0.88; df = 2; *p* = 0.65) or T30 (Friedman χ2 = 1.08; df = 2; *p* = 0.58).

For the cycling performance (Table 3), a Friedman ANOVA indicated no significant differences between Condition neither before (Friedman χ2 = 3.13; df = 2; *p* = 0.21) nor at the end of the performance (Friedman χ2 = 2.60; df = 2; *p* = 0.27). The pairwise comparisons within condition revealed a significant increase of TS’measures from T0 to T20 in NO-VEST (T = 0.00, Z = 2.02, *p*<0.05), but no significant differences for either ICE-VEST (T = 3.50, Z = 1.78, *p* = 0.08) or MEN-VEST (T = 1.00, Z = 1.75, *p* = 0.08) (data not represented graphically).

During the warm-up, no significant differences were found for the TC values from T0 to T30 whether for ICE-VEST (Friedman χ2 = 2.11; df = 6; *p* = 0.91), MEN-VEST (Friedman χ2 = 2.11; df = 6; *p* = 0.91) and NO-VEST (Friedman χ2 = 9.86; df = 6; *p* = 0.13) (see Table 3). No inter-condition differences were found for T0 (Friedman χ2 = 0.67; df = 2; *p* = 0.72), T5 (Friedman χ2 = 1.40; df = 2; *p* = 0.50), T10 (Friedman χ2 = 0.50; df = 2; *p* = 0.78), T15 (Friedman χ2 = 0.67; df = 2; *p* = 0.72), T20 (Friedman χ2 = 1.33; df = 2; *p* = 0.51), T25 (Friedman χ2 = 0.35; df = 2; *p* = 0.84) or T30 (Friedman χ2 = 0.50; df = 2; *p* = 0.78).

For the cycling performance (see Table 3), a Friedman ANOVA indicated no between-condition differences neither before (Friedman χ2 = 0.67; df = 2; *p* = 0.72) nor at the end of the performance (Friedman χ2 = 1.88; df = 2; *p* = 0.39). The pairwise comparisons within condition revealed no difference of TC’measures from T0 to T20 for ICE-VEST (T = 7.00, Z = 0.74, *p* = 0.46), MEN-VEST (T = 4.00, Z = 0.37, *p* = 0.72) and NO-VEST (T = 0.00, Z = 1.34, *p* = 0.18).

## Discussion

The aim of this study was to determine in a tropical climate (1) whether combined internal pre-cooling with menthol and external pre-cooling with or without menthol during a warm-up would improve the cycling performance, perceptions (i.e., thermal environment, RPE) and core temperature of athletes more than only internal pre-cooling with menthol, and (2) in the mixed conditions, whether adding menthol to the external pre-cooling would further improve the cycling performance, perceptions and core temperature. No between-condition differences were found for physiological parameters, perception of exertion, thermal comfort and thermal sensation during either the warm-up or the performance. Nevertheless, an increase of the thermal sensation was observed during the performance only in NO-VEST condition.

This study failed to show between-condition differences for the cycling performance for either the total distance traveled nor the distance traveled per 2-min block. Regarding the latter, a period effect was observed, suggesting a variation in pace during the cycling performance trial. Visually only, the Fig 1 shows more variation in pace in the NO-VEST condition than the mixed conditions, suggesting a better consistent performance with the both external cooling methods. These could have had an impact on effort regulation leading to the subject to provide a homogeneous and constant effort over time. Furthermore, the Fig 1 also allows to notice that the evolution of the curves of the two mixed conditions are relatively close.

A study with cyclists focused on the effects of a precooling during an active warm-up on a short distance time trials in hot conditions and has shown, at the end of the warm-up, that an upper body precooling (head, neck and torso) enhances cycling performance time and limits increase of skin temperature, mean body temperature, and thermal comfort ratings [38]. Our study failed to show the effectiveness of precooling during the warm-up on short-distance cycling performance, cooling rate and perceptions, probably due to some differences in the protocol. Indeed, as our warm-up time was longer (30 min versus 20 min) and our performance task more demanding (time trial events versus 65% $\dot{V}O2peak$), our precooling methods, which also differed (ice vest with or without menthol + internal menthol cooling versus ice vest + head and neck), were not sufficiently effective in such conditions, particularly faced with the tropical humid climate that impact the evaporation process by limiting the dissipation of the heat load.

In our protocol, it was expected that (1) the combination of internal and external cooling, and/or (2) the addition of menthol to this combination, would improve the thermal perception and the cooling rate during warm-up, and improve the distance covered and the perception of effort during this performance. Given the more demanding and longer warm-up on the one hand, and the performance of a task in a more humid and ecological environment on the other hand, it is possible that the skin surface directly covered by the precooling was insufficient to improve subsequent performance and the thermal sensation, especially considering the physiological responses warm-up-induced. Indeed, in a review on the precooling methods (Rodriguez et al.), the body surface exposed, its sensibility and the time of application need to be considered. In addition, the neck, the head and the face, due to the proximity to the thermoregulatory center of the central nervous system [39], are regions of high alliesthesial thermosensitivity [40], and, despite constituting only a small portion of the total skin surface area, dominate whole-body temperature perception [41]. These suggest that, in order to improve subsequent performance in the heat when a demanding warm-up is performed, future active investigations should focus on the head and/or the neck with a particular attention to perceptions of the thermal environment (in addition of the core body temperature).

Regarding menthol, it was expected that the addition of this compound of Mentha to the external pre-cooling in the mixed conditions (ICE-VEST and MEN-VEST) would improve the cycling performance, perceptions and cooling rate. As this study failed to show differences between the mixed conditions for these parameters during either the warm-up or the performance, it seems that the addition of menthol in skin precooling does not optimize the combined technique used here, i.e. internal pre-cooling with menthol and external pre-cooling. A study, with two independent variables (i.e., (1) with or without menthol, and (2) ambient or cold temperature), focused on the effects of a torso application of a 4% menthol solution during a running performance (i.e., 10 km) in tropical climate [42]. Compared to the control condition (water at ambient temperature), run performances were all improved but no differences were observed between the three experimental conditions either in performance or temperature. Although our study focuses on precooling, but given the requirement of the warm-up (in intensity and duration), our results confirms the fact that combining traditional external cooling with menthol do not add value for a performance in hot and humid conditions. Another explanation would be that the ingestion of the cold menthol water in both mixed conditions could have a stronger impact than that of the cooling vest, not allowing sufficiently discriminate the potential effects of cutaneous application (cold water and menthol+cold water) by wearing the vest. Further data are needed to (1) to isolate the effect of ingestion (internal cooling) from that of application to the skin (external cooling) and thus, determine whether an internal pre-cooling with menthol is sufficient alone for short distance time trials (e.g., by comparing with no pre-cooling and/or internal pre-cooling without menthol), and (2) investigate the add-value of the menthol on skin external application during an active warm-up in hot and humid conditions.

Several studies have suggested an effect of the duration of the pre-cooling and/or the duration of the rest interval [18, 21, 43, 44]. For example, a longer pre-cooling period (i.e., >30 min) would be beneficial for subsequent performance [43, 44] and a better pre-cooling effect on performance was observed with cold menthol beverage drunk over 1 hour instead of 30 min [18]. Naito et al. showed that a long rest interval (i.e., 20 min rest versus 5 min rest) after internal pre-cooling by ice ingestion had the greatest positive effect on exercise capacity (cycling performance time to exhaustion) in a hot and humid environment [21]. In our study, the transition between the end of the 30-min warm-up and the beginning of the cycling performance was 5 min. Despite the combination of two cooling strategies (internal and external), the transition time after a very active warm-up (reflected by the continuous increase of the RPE from T5 to T25 before a decrease at the end of the warm-up) was probably not long enough to allow the cyclists to benefit from the effects of the different pre-cooling strategies that are supposed to limit the heat storage capacity and the increase in the thermal perception.

Although no condition differences were found during either the warm-up or the cycling performance, an increase of the thermal sensation was observed during the cycling performance only in the NO-VEST condition, without any modification of the comfort. At the end of the performance, the participants seem to have perceived the environment as being warmer in the condition in which they did not wear the cooling vest during the warm-up. Compared to the “static” warm-up, achieving a cycling performance in an ecological situation (e.g., a velodrome) has the advantage of benefiting from the movement of air allowing an optimization of the convection process, thus enabling evaporative thermolysis capabilities [45]. The absence of an increase in the thermal sensation at the end of the cycling test under conditions where the subjects wore a cooling vest during warm-up, with or without menthol, suggests a beneficial effect of wearing a cooling vest on thermal perception although it had no effect on performance: the thermal sensation may have engaged the athlete to maintain a greater intensity of exercise and for longer leading to, visually on Fig 1, a homogeneous and constant effort over time as discussed previously. Given all these, it could be that the final cycling task of 20 min was not long enough to identify the possible benefits of wearing a cooling vest in the mixed conditions. Indeed, a study on a 2000-m rowing female performance showed no improvement in performance after a (passive) pre-cooling period, probably due to the short exercise time [46]. Rodriguez et al. concluded in their review that better results from the application of pre-cooling were observed in prolonged physical exercise than in short bouts [28]. In 2002, Marino also questioned the benefit of pre-cooling strategies for short-duration exercises and emphasized the likely effectiveness for endurance exercise of up to 30–40 minutes [47]. In our study, the exercise was probably not long enough to observe an effect of pre-cooling on performance.

Finally, the thermal sensation analyses revealed a continuous increase over time in all conditions before the decrease at the end of the warm-up without affecting the thermal comfort. These results are probably due to the metabolic heat production generated by the exercising muscles during the warm-up without condition difference.

Although caution is needed given the small sample size, the lack of condition differences during the warm-up and the cycling performance could suggest that the mixed interventions may not have been discriminating enough to limit the increase in the perception of exertion during the warm-up and to positively impact the cycling performance, the latter being probably too short.

Regarding the period, the analyses showed a continuous increase over time in all conditions before the decrease at the end of the warm-up. These results confirmed that the participants correctly performed the warm-up, finding it increasingly more difficult as the imposed intensity increased from T0 to T25.

The HR analyses showed a clear increase over time during the warm-up and performance in all conditions, attesting to the exercise-induced increase in cardiac load. Although an interaction effect was observed during the warm-up, suggesting pattern differences between the conditions, caution is needed due to the small sample size, especially that no effect of menthol has been shown on heart rate during sport performance [48]. Regarding the cooling rate, no difference between conditions was found for ∆Tgi during warm-up or the cycling performance. Despite a large interindividual variation and a small sample, it is interesting to visually note on the Fig 2 temperature differences between conditions. During the warm-up (Fig 2A), the temperature difference was greater in NO-VEST condition, assuming a greater increase in temperature without a cooling vest, whereas the differential was not very emphasized in the mixed conditions with a vest. Conversely, it should be noted that the temperature difference during the performance appeared to be greater in the mixed conditions (i.e., conditions with cooling vests during the warm-up). Although caution is needed with these data, the athletes, due to the cooling vests worn during the warm-up, could have benefited from a greater amplitude of temperature change leading them to maintain a better consistent performance (as previously discussed with the Fig 1).

A study on the thermoregulatory influence of a cooling vest concluded that this intervention did not rapidly reduce elevated body temperature and did not improve the cooling rate [49]. In our study, the cooling vest (external pre-cooling with or without menthol) was associated with the ingestion of cold menthol water (internal pre-cooling) during the warm-up in two out of the three conditions (ICE-VEST and MEN-VEST). As the third condition was only an internal pre-cooling with ingestion of cold menthol water during the warm-up, our results failed to show the effectiveness of these mixed pre-cooling strategies on the change in Tgi. Moreover, some authors have highlighted the positive impact of a long rest between pre-cooling and exercise on the time to exhaustion and the core temperature [18, 21]. In our study, the cyclists had only a 5-min transition between the warm-up on the cycle trainer and the starting line in the velodrome. This transition was probably too short to allow the pre-cooling strategies to lower the core temperature sufficiently before the cycling performance began, and thus it could not enhance the total distance traveled over 20 min. Also, evaporation is hindered in a hot and wet environment [4], and a humid layer added to an already tropical condition, even if it was cooled (i.e., the regularly refreshed cooling vest in ICE-VEST and MEN-VEST conditions), may have impacted the evaporation process, limiting the dissipation of the heat load and leading to no improvement in the cooling rate.

The main strength of this study was to have considered methods of precooling during a demanding warm-up, which has not often been tested in the literature. Having considered this reinforces the ecological aspect of this study. The counterpart is that a small number of subjects could be recruited due to the demanding selection criteria in a small area (i.e., heat-acclimatized well-trained male road cyclists competing regularly in elite road races). Another important limitation of this study is to have presented physiological parameters in the form of a cooling rate, thus limiting the possible appearance of variations and their interpretation. Given all these, future investigations, which will have to consider wind speed in addition, are needed to recruit a very large number of subjects in order to clarify the effectiveness of these mixed techniques (an internal pre-cooling method with menthol associated with external pre-cooling with or without menthol during warm-up) and to discriminate the added value of menthol−on−skin pre-cooling during a performance longer than 20 min. This would be particularly useful when the warm-up is performed on a cycle trainer before a long−duration cycling race.

## Conclusion

Results of this study failed to show improvements in performance (total distance), physiological parameters (cooling rate, HR) and perceptions (RPE and thermal perceptions) according the precooling conditions. Nevertheless, an increase of the thermal sensation was observed during the performance only in NO-VEST condition. The absence of a modification of the thermal sensation at the end of the cycling test under the mixed conditions (ICE-VEST and MEN-VEST) could suggest a beneficial effect of wearing a cooling vest on thermal sensation although it had no effect on performance. Therefore, other methods to improve performance of well-trained male cyclists in a velodrome during a short distance time trials in hot and humid conditions should be considered. Thus, further data are needed to develop this, but also to test the impact of the mixed techniques using regions of high alliesthesial thermosensitivity (neck, head, face) and during longer performances.

## Supporting information

S1 Data (XLSX)
